# N-Acetylcysteine (NAC) Ameliorates Lipid-Related Metabolic Dysfunction in Bone Marrow Stromal Cells-Derived Adipocytes

**DOI:** 10.1155/2018/5310961

**Published:** 2018-10-17

**Authors:** Marco Raffaele, Ignazio Barbagallo, Maria Licari, Giuseppe Carota, Giuseppe Sferrazzo, Mariarita Spampinato, Valeria Sorrenti, Luca Vanella

**Affiliations:** Department of Drug Science, Biochemistry Section, University of Catania, Viale A. Doria 6, 95125 Catania, Italy

## Abstract

Recent experimental data suggest that fatty acids and lipotoxicity could play a role in the initiation and evolution of metabolic bone diseases such as osteoporosis. A functional bone marrow adipose tissue (BMAT) may provide support to surrounding cells and tissues or may serve as a lipid reservoir that protects skeletal osteoblasts from lipotoxicity. The present study examined the effect of N-acetylcysteine (NAC), a powerful antioxidant and precursor of glutathione, commonly used to treat chronic obstructive pulmonary disease, on triglycerides accumulation in bone marrow stromal cells-derived adipocytes. Quantification of Oil Red O stained cells showed that lipid droplets decreased following NAC treatment. Additionally, exposure of bone marrow stromal cells (HS-5) to NAC increased adiponectin, PPAR*γ*, HO-1, and SIRT-1 and increased beta-oxidation markers such as PPAR*α* and PPAR*δ* mRNA levels. As there is now substantial interest in alternative medicine, the observed therapeutic value of NAC should be taken into consideration in diabetic patients.

## 1. Introduction

Obesity, diabetes, and metabolic syndrome are linked to abnormal bone homeostasis. The balance between bone formation and resorption is compromised in diabetes, which may contribute to the high risk of fractures in diabetic patients [1–4].

Osteoporosis is a metabolic bone disease associated with a dysregulated bone remodeling resulting from an alteration of the balance between bone formation and bone resorption but although the association between osteoporosis and type I diabetes (T1D) is well established [5], the reported association between osteoporosis and type II diabetes (T2D) is less clear. Chronic hyperglycemia, observed in T2D, accelerates the nonenzymatic process of protein glycosylation, resulting in the formation and accumulation of advanced glycation end-products which contribute to skeletal fragility [6, 7].

Accumulative evidence has indicated that T2D is associated with a reduced bone turnover and consequently a poor bone quality caused by lower levels of bone formation and resorption [8, 9].

Interestingly, an increased bone marrow adiposity (BMA) has been reported in T1D and T2D; however, in T1D, it was observed a decrease in bone mass, whereas T2D is characterized by no change or higher bone mass and paradoxically increased risk of bone fractures [10, 11]. In the last decade, there has been increasing interest in the formation, function, and potential endocrine roles of bone marrow adipose tissue (BMAT) [12–14].

BMAT develops postnatally and accounts for 50–70% of bone marrow volume in healthy adult humans. Historically BMAT was considered as an inert type of fat which accumulates in the bone marrow to fill empty space. Marrow adipocytes are no more regarded as simple filling cells, and their involvement in bone physiopathology is now considered. Indeed, BMAT can be considered a functional organ that can undergo pathologic changes and respond to diseases [15].

Furthermore, several studies support the notion that BMAT is significantly associated with skeletal health. In* vitro* studies have shown that adipocytes may directly influence osteoblast and osteoclast differentiation and function, through secretion of adipokines and free fatty acids, suggesting a direct effect of BMAT on bone turnover. BMAT adipocytes are situated in a unique microenvironment, surrounded by hematopoietic and skeletal lineage cells. Hyperglycemia has emerged as a major issue that threatens health and causes vascular and organ dysfunction. Recent reports indicate that hyperglycemia impairs bone marrow hematopoietic function and alters hematopoietic niche [16].

These observations suggest that BMAT may function to provide support to surrounding cells and tissues or may serve as a lipid reservoir, a storage site that protects skeletal osteoblasts from lipotoxicity or may exert systemic effects [17–19].

These findings have raised much interest in the potential role of BMAT supporting the concept that BMAT has an endocrine/paracrine function modulating marrow environment supporting bone remodeling and that this function is under similar regulatory axes as in peripheral adipose tissue.

Changes in bone fat metabolism may contribute to an increase in oxidative stress and in oxidized lipids associated with increased local inflammatory responses, resistance to anabolic effects of Wnt signaling, and osteoporotic bone formation. Bone mineral density and biochemical markers of bone turnover are adversely affected in individuals with diabetes. Indeed, glucose intolerance and saturated fatty acids are associated with the attenuation of bone remodeling and turnover in animal models of diet-induced obesity and diabetes [20, 21].

Increasing evidence confirmed that oxidative stress plays a role in the triggering of bone disease associated with diabetes [22].

Moreover, levels of reduced glutathione (GSH), which represents the major intracellular reducing molecule, are decreased in the bone marrow after induction of diabetes.

N-acetylcysteine (NAC) is a thiol compound that stimulates glutathione-S-transferase activity; it acts as a scavenger of free radicals and as an antioxidant by restoring the pool of intracellular GSH. NAC has been commonly used to treat chronic obstructive pulmonary disease [23, 24] but in addition to its antioxidant and anti-inflammatory properties, there is a growing interest in the beneficial effects of NAC for treating metabolic disorders. It has been shown that NAC supplementation suppresses fructose and high-sucrose diet-induced hyperglycemia and hyperinsulinemia and improves peripheral insulin sensitivity [25–27].

Additionally, Yamada et al. demonstrated that NAC markedly promoted the differentiation of osteoblastic cells and accelerated bone regeneration [28]. Despite some data demonstrating that NAC inhibits murine preadipocyte differentiation [29], its anti-inflammatory effects on human mature and hypertrophic bone marrow adipocytes are not known.

The goal of the present study was to determine whether NAC treatment affect lipid metabolism and triglycerides accumulation in bone marrow stromal cells-derived adipocytes.

## 2. Material and Methods

### 2.1. Differentiation of Human BMSC into Adipocytes

HS-5, human bone marrow stromal cells, were purchased from American Type Culture Collection (Rockville, MD, USA). After thawing, HS-5 cells were resuspended in DMEM, supplemented with 10% heat inactivated fetal bovine serum (FBS, Invitrogen, Carlsbad, CA, USA) and 1% antibiotic/antimycotic solution (Invitrogen), and plated in a 75 cm^2^ flask at a density of 1 to 2 × 10^4^ cells. The medium was replaced with adipogenic medium, and the cells were cultured for additional 19 days. The adipogenic media (Lonza, Basel, SW) consisted of complete culture medium supplemented with DMEM-high glucose (4.5 g/L), 10% (v/v) FBS, 10 *μ*g/ml insulin, 0.5 mM dexamethasone (Sigma-Aldrich, St. Louis, MO), 0.5 mM isobutylmethylxanthine (Sigma-Aldrich, St. Louis, MO), and 0.1 mM indomethacin (Sigma– Aldrich, St. Louis, MO). Medium was changed every 3 days. In our experiments human BMSCs were cultured in the presence of NAC (10 mM) which was added once for the last 3 days of differentiation.

### 2.2. Cell Viability Assay

HS-5 cells were seeded at a concentration of 2 × 10^5^ cells per well of a 96-well, flat-bottomed 200 *μ*l microplate. Cells were incubated at 37°C in a 5% CO_2_ humidified atmosphere and cultured for 24 h in the presence and absence of different concentrations (1, 10, and 100 mM) of NAC. Four hours before the end of the treatment time, 20 *μ*l of 0.5% 3-(4,5-dimethylthiazol-2-yl)-2,5-diphenyltetrazolium bromide (MTT) in phosphate buffered saline (PBS) was added to each microwell. After incubation with the reagent, the supernatant was removed and replaced with 100 *μ*l DMSO. The amount of formazan produced is proportional to the number of viable cells present. The optical density was measured using a microplate spectrophotometer reader (Thermo Labsystems Multiskan Italy) at *λ* = 570 nm.

### 2.3. Free Radical Scavenging Activity

The free radical scavenging activity of NAC was evaluated using the DPPH (2,2-diphenyl-1-picrylhydrazyl) test. An aliquot of MeOH solution containing NAC 10 mM was added to a daily prepared methanol DPPH solution (final concentration 0.1 mM). The optical density change at 517 nm was measured, 20 min after the initial mixing, using a microplate spectrophotometer reader (Thermo Labsystems Multiskan Italy). Rutin (50*μ*M) was used as reference. The scavenging activity was measured as the decrease in absorbance of the samples versus DPPH standard solution. The results were obtained from the average of three independent experiments and are reported as mean radical scavenging activity percentage (%) ± SD.

### 2.4. Measurement of HO Enzymatic Activity in HS-5 Cell Line

Total HO activity in the cell lysate was determined by measuring the bilirubin formation using the difference in absorbance at 464–530 nm as previously described [30].

Reaction mixtures (500 *μ*L) consisted of 20 mM Tris-HCl, pH 7.4, (1 mg/mL) cell lysate, 2 mg/mL biliverdin reductase, 1 mM NADPH, 2 mM glucose 6-phosphate (G6P), 1 U G6P dehydrogenase, and 25 *μ*M hemin. Incubations were carried out for 1 h at 37°C in a circulating water bath in the dark. Reactions were stopped by adding 1 volume of chloroform. After recovering the chloroform phase, the amount of bilirubin formed was measured with a double-beam spectrophotometer as OD464-530 nm (extinction coefficient, 40 mM/cm-1 for bilirubin). One unit of the enzyme was defined as the amount of enzyme catalyzing the formation of 1 nmol of bilirubin/ mg protein/h.

### 2.5. Oil Red O Staining

Staining was performed using 0.21% Oil Red O in 100% isopropanol (Sigma-Aldrich, St. Louis, MO, USA). Briefly, adipocytes were fixed in 10% formaldehyde, stained with Oil Red O for 10 minutes, and rinsed with 60% isopropanol (Sigma-Aldrich), and the Oil Red O was eluted by adding 100% isopropanol for 10 minutes and the optical density (OD) measured at 490 nm, for 0.5 sec reading. Lipid droplets accumulation was examined by using inverted multichannel LED fluorescence microscope (Evos, Life Technologies, NY).

### 2.6. RNA Extraction and qRT-PCR

RNA was extracted by Trizol reagent (Invitrogen, Carlsbad, CA, USA). First strand cDNA was then synthesized with Applied Biosystem (Foster City, CA, USA) reverse transcription reagent.

Quantitative Real-Time PCR was performed in 7900HT Fast Real-Time PCR System Applied Biosystems using the SYBR Green PCR MasterMix (Life Technologies). The primer sequences used are shown in Table 1.

The specific PCR products were detected by the fluorescence of SYBR Green, the double stranded DNA binding dye. The relative mRNA expression level was calculated by the threshold cycle (Ct) value of each PCR product and normalized with that of GAPDH by using comparative 2^–ΔΔCt^ method.

### 2.7. Statistical Analyses

Statistical significance (p<0.05) of differences between experimental groups was determined by the Fisher method for analysis of multiple comparisons. For comparison between treatment groups, the null hypothesis was tested by either single-factor analysis of variance (ANOVA) for multiple groups or the unpaired-test for two groups, and the data are presented as mean ± standard deviation (SD).

## 3. Results

### 3.1. Viability Assessment

**Figure 1:**
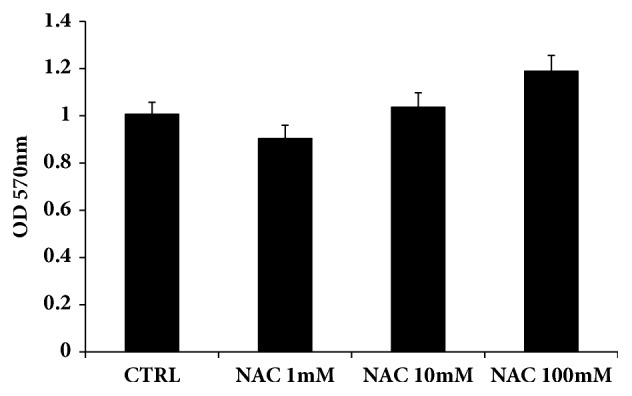
The cytotoxicity of NAC (1, 10, 100 mM) on HS-5 cells. Cells were incubated with NAC for 24 h and cell viability was assessed using an MTT assay. Values represent the mean ± SD of 4 experiments.

The absence of cytotoxicity of NAC was assessed by the MTT assay, a widely recognized in vitro preliminary screening able to individuate time and concentration-dependent toxic effect on mammalian cell vitality and growth. After a 24 h incubation period to various concentrations of NAC (1, 10, 100 mM), MTT assay (Figure 1) did not reveal any cytotoxic effect of NAC against HS-5 bone marrow stromal cells.

### 3.2. Antioxidant Activity of NAC

**Figure 2:**
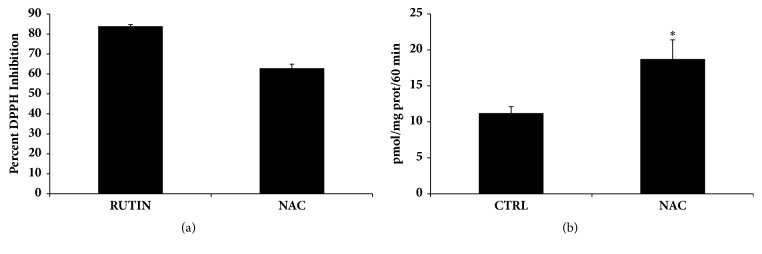
(a) DPPH radical scavenging activity of NAC 10 mM compared with the standard antioxidant Rutin 50*μ*M. Results are expressed as percent DPPH inhibition. The data shown are the mean from three independent experiments. (b) HO-1 activity in HS-5 cell lysate in absence and in presence of NAC 10 mM. Values, expressed as pmol bilirubin/mg protein/60min, represent the means ± SD of four experiments performed in triplicate. Significant versus untreated control cells: ^*∗*^p < 0.05.

Because of its odd electron, DPPH gives a strong absorption band at 517 nm in visible spectroscopy (deep violet color). As this electron becomes paired off in the presence of a free radical scavenger, the absorption vanishes and the resulting decolorization is stoichiometric with respect to the number of electrons taken up. As shown in Figure 2(a), NAC exhibited DPPH free radical scavenging activity in a cell-free system. As DPPH is a synthetic radical, we also investigated the antioxidant capacity of NAC by measuring heme oxygenase derived bilirubin production in a cellular system. Under our experimental conditions, exposed HS-5 cells to 10 mM NAC for 24 hr showed a significant increase in heme oxygenase activity compared to untreated control cells (Figure 2(b)).

### 3.3. Effect of NAC on Adipocyte Lipid Metabolism

**Figure 3:**
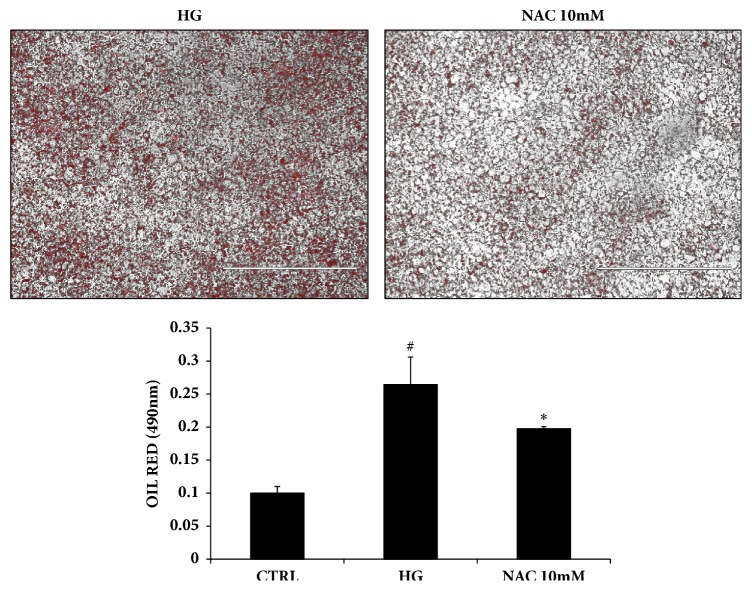
Representative Oil Red O staining of HS-5 cells in presence and absence of NAC (10mM). Lipid content was measured as the relative absorbance of Oil Red O at day 19 after inducing adipogenesis as described in materials and methods (mean ± SD, ^*∗*^*p* < 0.05 versus high glucose). Values represent the means ± SD of 4 experiments performed in triplicate.

**Figure 4:**
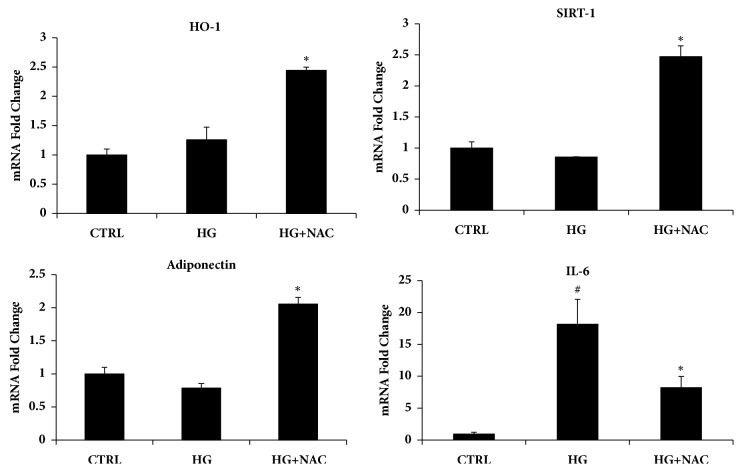
Analysis of gene expression by Real-Time PCR of HO-1, SIRT-1, adiponectin, and IL-6. All values are expressed as mean ± SD of four experiments (*n* = 4) in duplicate (^*∗*^p < 0.05 versus high glucose; ^#^p < 0.05 versus control).

We examined the effect of NAC on lipid accumulation after 19 days, using standard culture conditions by measuring Oil Red O stained lipid droplet area (Figure 3). Quantification of Oil Red O stained cells showed that lipid droplets decreased following NAC treatment. In addition to that, the expression of SIRT-1 and the antioxidant enzyme HO-1 in the presence of NAC significantly increased after 19 days (Figure 4), which was consistent with our previous results showing HO-1 induction decreased lipid droplets [31–33].

**Figure 5:**
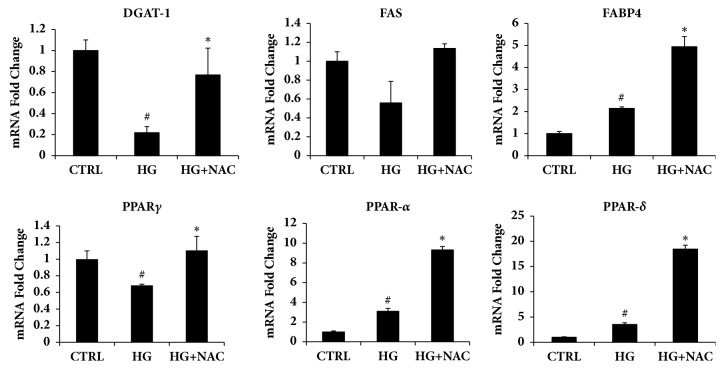
Analysis of gene expression by Real-Time PCR of lipogenic pathway. All values are expressed as mean ± SD of four experiments (*n* = 4) in duplicate (^*∗*^p < 0.05 versus high glucose; ^#^p < 0.05 versus control).

To further examine the mechanism by which NAC regulates lipid metabolism, we measured DGAT1, FABP4, FAS, PPAR*γ*, PPAR*α*, PPAR*δ*, and adiponectin mRNA levels in adipocytes (Figures 4 and 5). Interestingly, as seen in Figure 5, DGAT1 mRNA expression was found to be significantly decreased in mature adipocytes compared to control cells and this effect was reversed by concurrent treatment with NAC. Our results further demonstrated that the expression of PPAR*γ* was significantly downregulated after 19 days of differentiation and this effect was negated by the treatment with NAC. Also our data showed that FAS levels were not significantly altered in all groups but FABP4 levels were increased by NAC treatment. Further, our results on beta-oxidation markers, such as PPAR*α* and PPAR*δ*, showed that NAC treatment increased both mRNA levels.

Adipose cell enlargement is associated with increased secretion of cytokines, which impairs the differentiation of preadipocytes and reduces adiponectin secretion. We examined the levels of IL-6 in NAC-treated HS-5 and found that IL-6 levels were significantly decreased. In contrast, adiponectin levels were increased following NAC treatment when compared to controls at day 19 (Figure 4).

## 4. Discussion

Although there is extensive literature on the role of visceral, subcutaneous, and brown adipose depots in the development of insulin resistance and type II diabetes, few studies have investigated the function of BMAT as an adipose storage organ [34].

Studies are underway to examine the role of impaired glucose metabolism and its associated hyperglycemia and hyperinsulinemia on the biological functions of BMSC [35].

Evidence collected from animal and human studies indicate that impairment in adipose tissue metabolism correlates with decreased bone quality.

A recent clinical study demonstrated very clearly that adipocyte size is associated with vertebral fractures supporting the hypothesis that large adipocytes are inflamed and produce several proinflammatory cytokines which affect bone quality as well as progenitor cells [36–38].

Currently, there are several ongoing studies examining the potential positive effects of BMAT on bone mass. A recent and innovative hypothesis suggest that BMAT can be targeted to treat bone disease since BMAT could support bone formation and remodeling. Marrow fat may participate in lipid metabolism by clearing and storing circulating triglycerides, thereby providing a localized energy reservoir for osteogenesis during bone fracture healing.

Recent experimental data suggest that FA and lipotoxicity could play a role in the initiation and/or evolution of metabolic bone diseases such as osteoporosis [39].

Although in small quantities relatively to white adipose tissue, marrow adipocytes produce leptin and adiponectin. Thus, it is reasonable to believe that BMAT has a local endocrine/paracrine function modulating marrow environment supporting bone remodeling and that this function is under similar regulatory axes as in peripheral fat.

Although NAC is a well-known thiol compound used in chronic obstructive pulmonary disease because it possesses a free sulfhydryl group through which it reduces disulfide bonds conferring antioxidant effects, NAC possess other beneficial effects on glucose and lipid metabolism [40].

Yang et al. reported that NAC administration partially reduces plasma triglycerides, total cholesterol, and LDL levels while increasing the HDL level in rats fed a high fat diet [41].

Short-term NAC administration in drinking water prevented high-sucrose induced weight gain and dyslipidemia. Collectively, these studies show that NAC is capable of restoring dysregulated glucose and lipid metabolism, suggesting its potential application in metabolic disturbance.

Our results demonstrate that NAC exhibits an interesting antioxidant activity in cell-free and in cell systems. It was able to quench the synthetic DPPH radical and exhibited an increase in heme oxygenase activity as shown by rise in bilirubin formation.

In adipose tissues, induction of HO-1 has been shown to reduce body weight, decrease proinflammatory cytokines, and increase PGC-1*α* mediated thermogenesis, thereby increasing energy uptake and the stimulation of mitochondrial FA oxidation [42–45].

To investigate the effects of NAC on human bone marrow adipocytes, HS-5 cells were exposed to NAC added to the differentiation medium for three days. HS-5 accumulated lipid droplets following exposure to the differentiation medium; however, consistent with previous reports on murine cells, lipid accumulation in the presence of NAC was reduced compared to untreated cells. To assess whether the reduced lipid droplets formation, induced by NAC, could be explained by altered activation of typical key markers of mature adipocytes, PPAR*γ*, DGAT1, FABP4, FAS, and adiponectin levels were investigated. Figures 4 and 5 show that NAC increases DGAT1, FABP4, and adiponectin levels. The latter represents a hormone with insulin-sensitizing, anti-inflammatory, and antiapoptotic functions released by functional healthy adipocytes.

PPAR*γ* represents the master regulator of adipogenesis and increased PPAR*γ* signaling in mature adipocytes is linked to improved insulin sensitivity [46]. Recently, it has been shown that specific modulators of PPAR*γ* activity, specifically those which protect Ser112 phosphorylation, induce beige-like profile in BMAT and in peripheral white adipose tissue and have either no or positive effect on bone, in contrast to thiazolidinediones which dephosphorylate Ser112 and have negative effects on bone structure [47, 48]. Our unexpected results on DGAT1 might support the concept that the function of DGAT1-mediated FA reesterification is not to preserve TG mass but instead protects the endoplasmic reticulum from lipotoxic stress and associated adipose tissue inflammation. Indeed, DGAT levels are decreased in mature adipocytes (Figure 3), suggesting that hypertrophic adipocytes lose the esterification function essential to reesterificate free fatty acids which represent the main cause of insulin resistance. In support of that, Dgat1-deficient mice have abnormal bone architecture and increased levels of osteoclast differentiation [49]. In addition to that, low FABP4 expression in adipose tissue could lead to less free fatty acids transport to *β*-oxidation, resulting in a fatty acids accumulation, which may exceed the adipose tissue storage capacity, resulting in excess fat “overspilled” to nonadipose tissues [50].

Further beneficial effects mediated by NAC are associated with an increase in mRNA levels of genes responsible for fatty acid oxidation and HO-1 induction. Indeed, NAC cause a strong induction of PPAR*α* (8-fold increase) and PPAR*δ* (15-fold increase) compared to untreated cells. These data support the beneficial interaction between bilirubin and PPAR alpha [51].

Several studies demonstrated that bilirubin treatment decreased body weight and body fat and increased lean body mass in wild-type but not in PPAR*α* knockout mice [52, 53].

**Figure 6:**
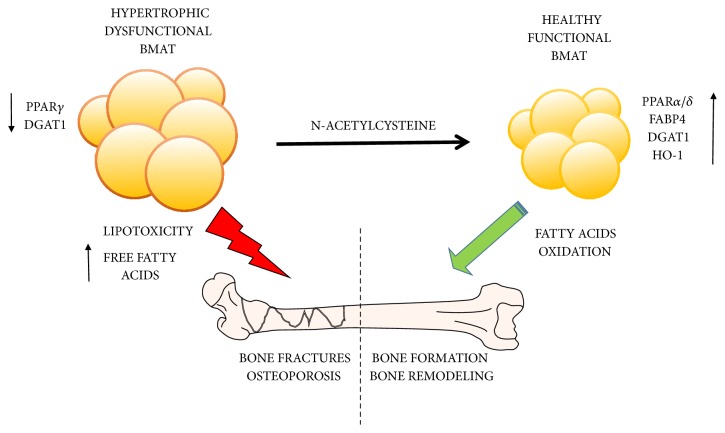
Proposed mechanisms demonstrating the role of NAC in the regulation of lipid metabolism. NAC restores the function of BMAT which may protect skeletal osteoblasts from lipotoxicity.

In conclusion, we show here in a cell-based model of adipogenesis that NAC entails reduced lipid accumulation comprising smaller healthier adipocytes, reduced inflammation, and improved adipokine secretion (Figure 6). These results provide direct evidence to support the use of NAC supplementation as an effective drug metabolic disorders.

## Figures and Tables

**Table 1:** PCR primers used in this study.

**Gene **	**Primer Forward**	**Primer Reverse**
**Adiponectin**	AGGCTTTCCGGGAATCCAAG	CGCTCTCCTTCCCCATACAC
**DGAT1**	CGCGGACTACAAATGGACGA	AACCAGTAAGACCACAGCCG
**FABP4**	AAACTGGTGGTGGAATGCGT	GCGAACTTCAGTCCAGGTCA
**FAS**	CGGAGGCATCAACCCAGATT	GATGGTGGTGTAGACCTTCCG
**GAPDH**	AGACACCATGGGGAAGGTGA	TGGAATTTGCCATGGGTGGA
**IL-6**	CTTCTCCACAAGCGCCTTCG	CTGGCATTTGTGGTTGGGTC
**PPAR** **α**	AAGAGCTTGGAGCTCGGC	TGAAAGCGTGTCCGTGATGA
**PPAR** **δ**	GGGACAGGCTGATGGGAAC	TGAACACCGTAGTGGAAGCC
**PPAR** **γ**	AGAGTACGTGGGAGAAATGAC	GATGGCCACCTCTTTGCTCT
**SIRT-1**	TGATTGGCACAGATCCTCGAA	AAGTCTACAGCAAGGCGAGC
**HO-1**	GTGCCACCAAGTTCAAGCAG	CACGCATGGCTCAAAAACCA

## Data Availability

The data used to support the findings of this study are available from the corresponding author upon request.
